# Doppler echocardiography and myocardial dyssynchrony: a practical update of old and new ultrasound technologies

**DOI:** 10.1186/1476-7120-5-28

**Published:** 2007-09-06

**Authors:** Maurizio Galderisi, Fabio Cattaneo, Sergio Mondillo

**Affiliations:** 1 Division of Cardioangiology, Department of Clinical and Experimental Medicine, Federico II University Hospital, Naples, Italy; 2 GE Healthcare, Milan, Italy; 3 Cardiology, University of Siena, Siena, Italy; 4 Laboratory of Echocardiography, Division of Cardioangiology with CCU, Department of Clinical and Experimental Medicine, Block 1, Federico II University Hospital, Via S. Pansini, 5, 80131 Naples. Italy

## Abstract

Morbidity and mortality rates are higher in patients with severe left ventricular (LV) systolic dysfunction and ECG-derived prolonged QRS interval than in those with normal QRS duration. QRS duration is currently used on the grounds that it reflects the presence of ventricular dyssynchrony. However, 30–40% of patients selected on the basis of a prolonged QRS do not receive benefit by cardiac resynchronization therapy (CRT) since they do not show any significant inverse LV remodeling and QRS duration does not accurately distinguish responders to CRT. Consequently, mechanical dyssynchrony (particularly intra-ventricular dyssynchrony) seems to be much more important than electrical dyssinchrony. Pre- and post-echocardiographic assessment should require the combination of conventional and specific applications ranging from M-mode and pulsed/continuous Doppler, to pulsed Tissue Doppler, the off-line analysis of colour Tissue Velocity Imaging, Strain Rate Imaging, and real time three-dimensional reconstruction However, there is not no consensus about the best approach and the best ultrasound parameter for selecting candidates to CRT and ECG representation of abnormal cardiac conduction still remains as the main criterion in guidelines. This review is a practical update of ultrasound methods and measurements of atrio-ventricular, inter-ventricular and intra-ventricular dyssynchrony and describes experiences which used either conventional Doppler echocardiography and more advanced techniques. By these experiences, the global amount of LV dyssynchrony seems to be critical: the greater intra-ventricular dyssynchrony, the higher the possibility of significant LV inverse remodeling. After CRT, it is necessary also to evaluate the optimal atrio-ventricular delay and ventricular-ventricular delay setting that maximizes LV systolic function.

## Background

Morbidity and mortality rates are higher in patients with severe left ventricular (LV) systolic dysfunction and ECG-derived prolonged QRS interval than in those with normal QRS duration [1]. **Bi-Ventricular Pacing **(**BIVP**) and **Cardiac Resynchronization Therapy **(**CRT**) have become additional treatment aimed to synchronizing biventricular activation and contraction in patients with severe chronic heart failure (CHF) associated with interventricular conduction delay. CRT is effective in improving functional capacity and degree of secondary mitral regurgitation [2-4] and, above all, in reducing the mortality in cases of refractory CHF. NYHA classes III-IV, a LV ejection fraction (EF) of ≤ 35%, a LV end-diastolic diameter > 30 mm/m^2 ^and a surface ECG derived QRS duration > 120 ms, together with a need for maximal pharmacological therapy, are considered from guidelines to select patients for CRT [5].

QRS duration is currently used on the grounds that it reflects the presence of ventricular dyssynchrony. However, 30–40% of patients selected on the basis of a prolonged QRS do not receive benefit by CRT since they do not show any significant inverse LV remodeling (a ≥ 15% reduction of LV end-systolic volume six months after device implantation) [6,7]. Furthermore, QRS duration does not accurately distinguish responders to CRT [8]. Although factors responsible for the absence of favourable response may be lead dislodgement or inappropriate location of LV lead, mechanical dyssynchrony (particularly intra-ventricular dyssynchrony) seems to be much more important than electrical dyssinchrony, and Doppler echocardiography should be widely used before and after implantation of a CRT device [9,10].

Pre- and post-echocardiographic assessment includes conventional and/or specific applications ranging from M-mode and pulsed/continuous Doppler, to pulsed Tissue Doppler, the off-line analysis of colour Tissue Doppler, Strain Rate Imaging (SRI), and real time 3-D reconstruction [9-13]. The different modalities of the transthoracic ultrasound approach are able to identify the 3 different kinds of mechanical dyssynchrony: 1. Atrio-ventricular dyssinchrony, 2. Inter-ventricular dyssynchrony, 3. Intra-ventricular dyssynchrony.

## 1. Atrio-ventricular dyssynchrony

**Figure 1:**
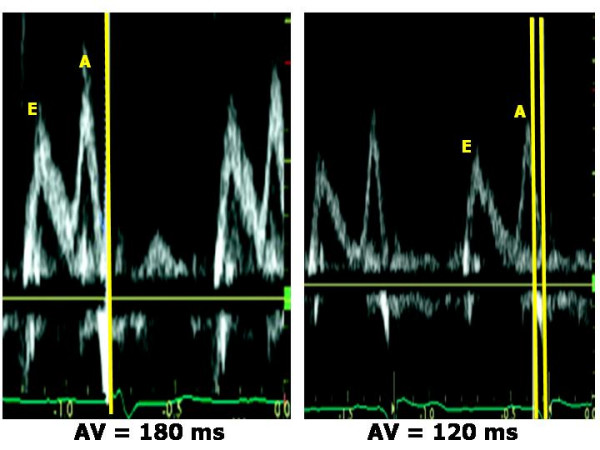
**Optimization of AV delay after CRT by pulsed Doppler mitral inflow pattern**. At programmed AV delay of 180 ms (upper panel) the closure of mitral valve occurs before the onset of ECG QRS. Optimizing AV delay at 120 ms (lower panel), the diastolic filling time is much longer and the duration of atrial filling is preserved. (Modified from Agler DA, *J Am Soc Echocardiogr *2007;20:76–90).

Atrio-ventricular (AV) dyssynchrony occurs in patients with dilated cardiomyopathy and first degree AV block and was the first objective of CRT by bicameral pacemakers in the early 1990s [14]. AV dyssynchrony reduces the duration of ventricular filling, thus inducing the appearance of diastolic mitral and tricuspid regurgitation and reducing stroke volume. The optimal AV delay after CRT is short, but a longer delay may be necessary in some cases (e.g., in the presence of concomitant inter-atrial delay). Optimizing AV delay is guided by a Doppler recording of transmitral inflow (***Mitral inflow method***) [11-13,15] which requires recording the mitral inflow pattern at a sweep speed of 100 mm/s, with the sample volume placed at the tips of the mitral leaflets; mitral valve closure must be clearly defined at the time of ECG R wave (Figure 1). The method cannot be applied in the presence of atrial fibrillation (no A velocity). Furthermore, when the AV delay is short (= 60–80 ms), the duration of A velocity may become shorter inducing less atrial contribution but total LV filling time increases.

## 2. Inter-ventricular dyssynchrony

**Figure 2:**
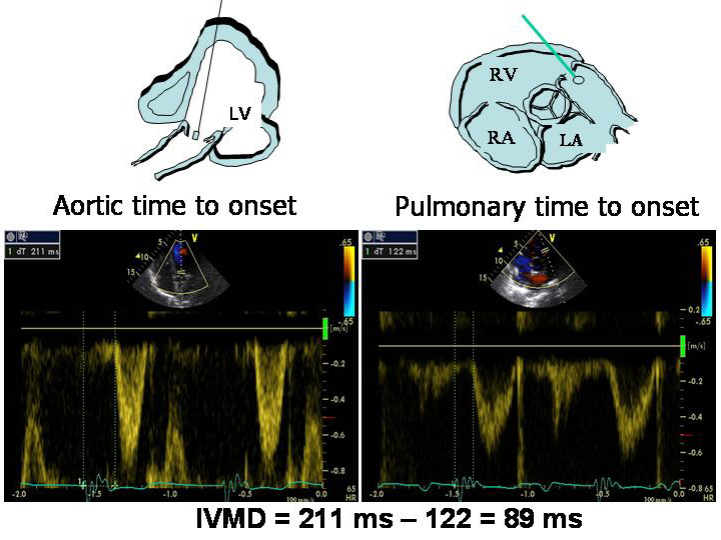
**Calculation of interventricular mechanical delay by standard Doppler method**. The time from ECG Q wave to onset of left ventricular outflow tract (= 211 ms) (left panel) is longer than the time occurring from Q to onset of right ventricular outflow tract (= 122 ms). The resulting inter-ventricular mechanical delay (IVMD) is of 89 ms, thus indicating a significant interventricular dyssynchrony.

Inter-ventricular dyssynchrony represents the discordance between the times of right ventricular (RV) and LV contraction. PW or CW Doppler images of aortic and pulmonary flow velocities are currently used to measure the ***inter-ventricular mechanical delay (IVMD)***, which includes recording of LV outflow tract (apical 5-chamber view) and RV outflow tract (parasternal short-axis view of the great vessels) and calculating the difference in time between ECG-derived Q wave onset and the onset of LV outflow and the time between the onset of Q and the onset of RV outflow [11,12] (Figure 2). These time intervals respectively reflect LV and RV pre-ejection period (PEP). IVMD values of > 40 ms and values of LV PEP of > 140 ms are considered pathological [16]. Doppler recording of LV and RV outflow velocities (sweep speed of 100 mm/s) requires appropriate gain and wall filter settings to visualize the opening and closing clicks. When using PW Doppler, the sample volume has to be placed proximally to the pulmonary and the aortic valve. The limitation of this method include the presence of pulmonary arterial hypertension and/or RV systolic dysfunction, which can prolong RV PEP, and a concomitantly impaired increase of LV pressure in very severe CHF.

Alternatively, pulsed Tissue Doppler can be used to determine IVMD by measuring the time from QRS onset to the peak myocardial systolic velocities (S_m_) of the RV free wall (tricuspid annulus) versus the same time of LV lateral mitral annulus (apical 4-chamber view) [9].

It is important to state that intraventricular dyssynchrony does not correlate with reverse LV remodeling after CRT, even when data from patients with and without coronary artery disease are evaluated separately [17-19].

## 3. Intra-ventricular mechanical dyssynchrony

Intra-ventricular dyssynchrony is characterized by either premature or late contraction of LV wall segments due to delayed electrical conduction [20]. It can be identified by means of simple M-mode, pulsed Tissue Doppler, or, better, by colour Tissue Velocity Imaging (TVI), SRI and 3-D echocardiography.

### M-mode

**Figure 3:**
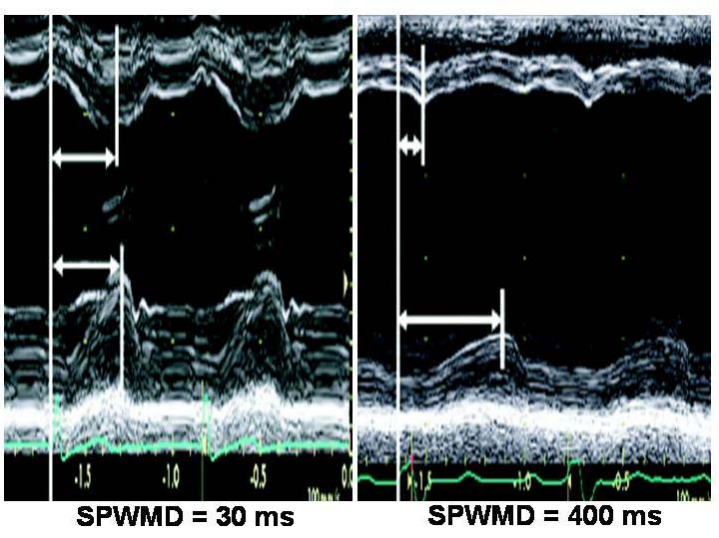
**Septal-to-posterior wall motion delay (SPWMD)**. SPWMD in a normal subject (left panel) and in a patient with CHF and left bundle branch block. (Modified from Agler DA et al, *J Am Soc Echocardiogr *2007;20:76–90).

Intra-ventricular mechanical delay can be determined on the basis of the simple M-mode-derived ***septal-to-posterior wall motion delay ***(***SPWMD***), i.e., the difference in timing of septal and posterior wall contraction [12,21,22]. The M-mode cursor is positioned perpendicular to the septum and posterior wall at the base of the left ventricle, in parasternal short-axis (or long-axis) view: SPWMD is the difference between the time from the onset of ECG-derived Q wave to the initial peak posterior displacement of the septum, and the time from the onset of QRS to the peak systolic displacement of posterior wall (sweep speed of 100 mm/s) (Figure 3). In the original experience of Pitzalis et al on 20 patients with advanced heart failure, a SPWMD (determined in parasternal short-axis view) of > 130 ms was considered pathological and SPWMD predicted inverse LV remodeling and long-term clinical improvement after CRT, with 100% sensitivity, 63% specificity and 85% accuracy [21,22]. The main advantages of this method correspond to the low-cost technique and the availability in all the echocardiographic machines. The limitations include the impossibility to measure SPWMD in patients with a poor acoustic window, previous septal or posterior wall myocardial infarction, or abnormal septal motion secondary to RV pressure or volume overload. In addition, M-mode can only visualize dyssinchrony of the anterior septum or posterior wall whereas other LV walls can be involved. Marcus et al have underlined the low feasibility of SPWMD (measured in parasternal long-axis view), which had also poor sensitivity (24%) and specificity (66%) in predicting the response to CRT of 79 heart failure patients [23].

**Figure 4:**
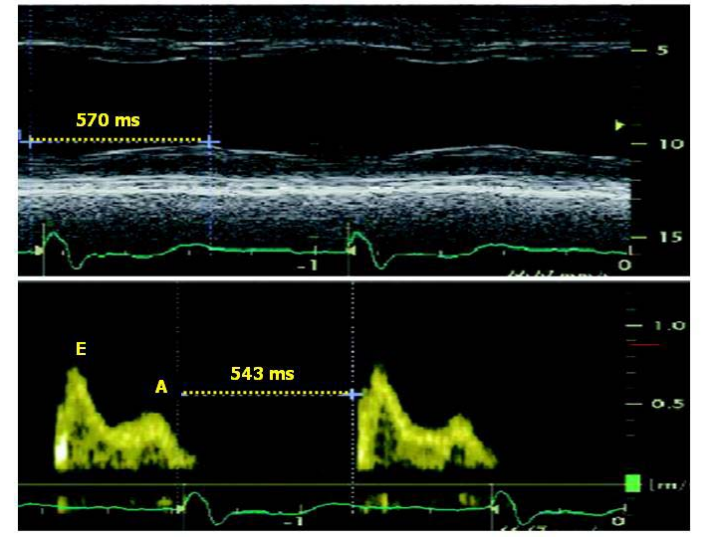
**Methodology for measuring lateral wall post-systolic displacement (LWPSD)**. It is measured as the difference of the time interval from QRS onset to maximal systolic displacement of the basal LV lateral wall (assessed by M-mode in the apical 4-chamber view) (upper panel) and the time interval from QRS onset to the beginning of transmitral E velocity (assessed by pulsed Doppler of mitral inflow) (lower panel). In this example, the positive value of the difference indicates the co-existence of segmental post-systolic contraction and diastolic relaxation. (Modified from Sassone B et al, *Am J Cardiol *2007;100:470–475).

A very recent method, reported by Sassone et al [24], determines ***lateral wall post-systolic displacement (LWPSD)***, measured as the difference of intervals from QRS onset to maximal systolic displacement of the basal LV lateral wall (assessed by M-mode in the apical 4-chamber view) and from QRS onset to the beginning of transmitral E velocity (assessed by pulsed Doppler of mitral inflow) (Figure 4). A positive LWPSD, i.e. a longer interval to maximal inward displacement of LV lateral wall than the interval to opening of the mitral valve, identifies a severe post-systolic contraction and has been demonstrated to be an independent predictor of CRT response in 48 patients with end-stage heart failure and left bundle branch block. Of interest, despite evaluating intra-ventricular dissynchrony based on delay of a single myocardial wall, LWPSD could predict CRT response. This may rely to the fact that, in the presence of left bundle branch block, the lateral wall is the latest to be activated, theoretically representing the optimal target for LV lead positioning. This method has not yet tested in patients without left bundle branch block and its diagnostic accuracy is unknown. In addition. it needs the identical heart rate when measuring the M-mode and pulse Doppler imaging.

### Pulsed (PW) Tissue Doppler

**Figure 5:**
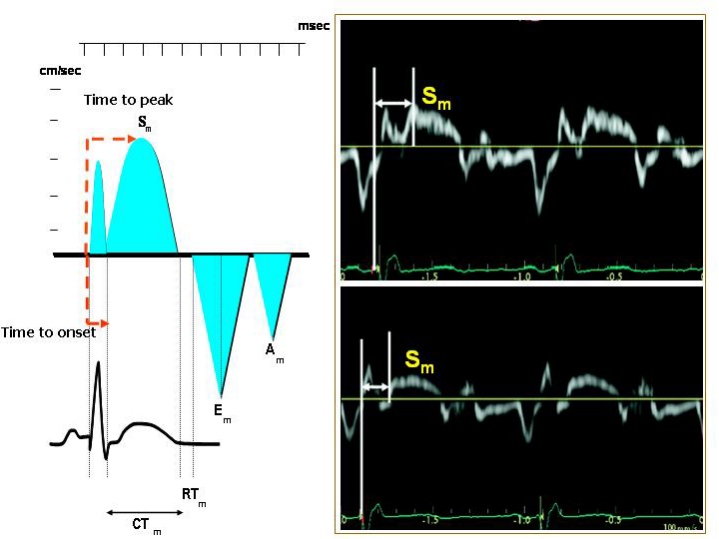
**Methodology for measuring pulsed Tissue Doppler derived time to peak Sm and time to onset Sm (left panel)**. In the right panel measurements of time to peak Sm (upper panel) and of time to onset Sm (lower panel) are depicted. Am = Myocardial atrial velocity, CTm = Contraction time, Em = Myocardial early diastolic velocity, RTm = Myocardial relaxation time, Sm = Myocardial systolic velocity. Mod from Agler DA et al, *J Am Soc Echocardiogr *2007;20:76–90.

Determining the time of myocardial systolic velocities (S_m_) is an important and easy echocardiographic means of assessing CHF before/after CRT. The advantage of PW Tissue Doppler is its excellent temporal resolution for measuring intraventricular mechanical dyssinchrony, and its availability in the majority of cardiac ultrasound systems. Various PW Tissue Doppler parameters have been proposed [25]. The most widely used measurements correspond to the time interval between the onset of ECG-derived QRS and the S_m _peak (= ***time to S*_*m *_*peak***) and the time interval between the onset of QRS and the onset of S_m _(= ***time to S*_*m *_*onset***), which correspond to LV PEP (Figure 5). Intra-ventricular mechanical delay has been defined for differences of > 65 ms of time to S_m _peak between LV segments [26]. Receiver-operator characteristic curve analysis demonstrated that this cut-off value yielded a sensitivity and specificity of 80% to predict clinical improvement and of 92% to predict LV reverse remodeling in 85 patients with end-stage heart failure, QRS duration > 120 ms, and left bundle-branch block [26].

Extensions of these method have been proposed by recording 2D imaging in the 4- 2- and 5-chamber apical views, in order to place PW Tissue Doppler sample volume in a specific myocardial segment and to measure Q to peak S_m _and/or Q to S_m _onset in various LV segments. The number of LV segments to be evaluated include mainly a 12-segment model (LV basal and middle segments in 4-, 2- and 5-chamber views) whereas LV apical segments are not considered reliable because of the basal-apical myocardial gradient own of Tissue Doppler. Technical refinements include the need to set the velocity scale of PW Tissue Doppler to display spectral velocities of 20 cm/s above and below the zero baseline because myocardial motion is characterized by low velocities. Spectral Doppler gain must be usually reduced, wall filters adjusted and spectral velocities recorded at sweep speed of 100 mm/s (during held respiratory expiration), in order to obtain the clearest delineation of S_m _onset and peak. Electromechanical delay has to be averaged over at least 3 cardiac cycles. The main limitation of PW Tissue Doppler corresponds to the impossibility of measuring the time intervals of different segments during the same cardiac cycle. It is also necessary to take into account that the S_m _recorded in apical views reflects LV longitudinal shortening and not circumferential contraction.

### Colour Tissue Doppler

**Figure 6:**
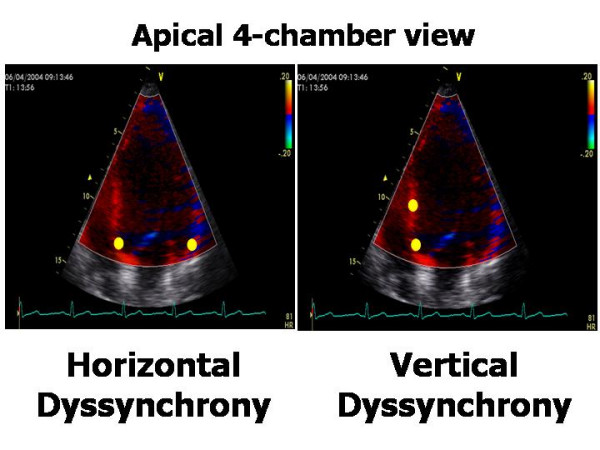
**Methodology for recording and measuring intra-ventricular horizontal and vertical dyssynchrony by off-line color Tissue Doppler techniques**. Horizontal dyssynchrony occurs between opposite walls. Vertical dyssynchrony is between different segments of the same wall.

**Figure 7:**
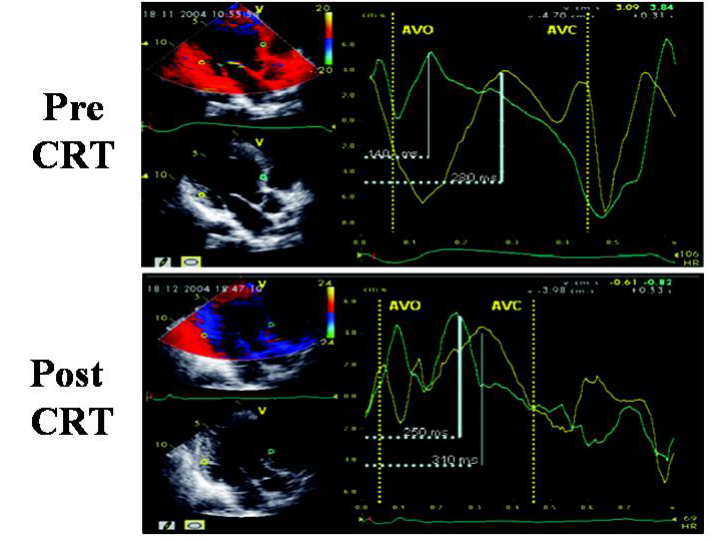
**Sample of time to peak (Ts) measured by off-line color TVI pre and post CRT at the level of anterior septum and postero-lateral wall, in apical 5-chamber view**. Aortic valve opening (AVO) and closure (AVC) are derived by previous placement of markers on the onset and the end of LV Doppler outflow. Pre-CRT Ts of postero-lateral wall (upper panel) is clearly delayed in comparison to Ts of anterior septum. Post-CRT the time duration of Ts between the two walls is clearly shortened (lower panel). (Modified from Innelli P et al, *Echocardiography *2006;23:709–716)

**Figure 8:**
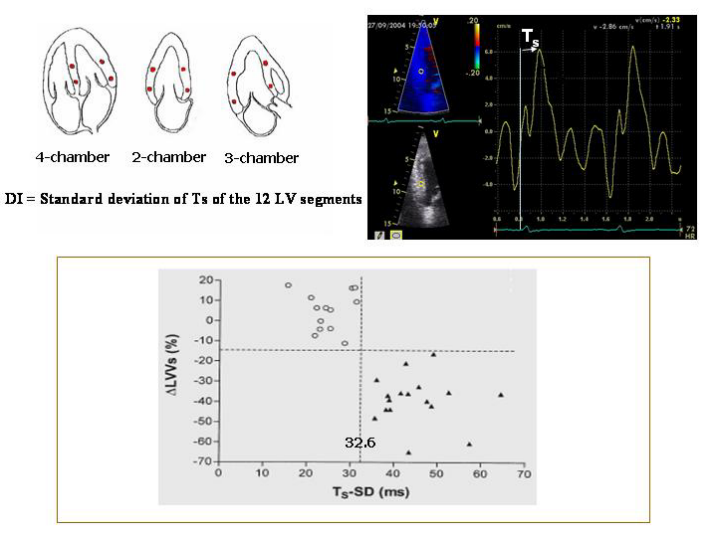
**Methodology of calculation of Dyssynchrony Index and is ability in predicting LV inverse remodeling**. In the upper panel methodology of calculation of Dyssynchrony Index, i.e., the standard deviation of Ts (Ts-SD) measured in the basal and mid-segments visualizable in the apical views. In the lower panel, Ts-SD shows the ability to predict an effective LV inverse remodeling after CRT (lower panel). Values of TS-SD > 32.6 (black triangles) predict an effective LV reverse remodeling (DLVVs = delta left ventricular end-systolic volumes) after CRT. Patients with pre-CRT values of Ts-SD < 32.6 (empty circles) do not present significant LV inverse remodeling at follow-up. (Modified from Yu CM et al, *Am J Cardiol *2003;91:684–688)

Off-line colour Tissue Doppler derived Tissue Velocity Imaging (TVI), Tissue Synchronization Imaging (TSI) and SRI can be used to assess intra-ventricular dyssynchrony in the longitudinal plane (apical views). The common advantages of these techniques is the possibility of measuring the dyssynchrony of opposite LV walls (= horizontal dyssynchrony) and of different segments of the same LV wall (= vertical dyssynchrony) in a given view, from the same cardiac cycle (Figure 6). Like to PW Tissue Doppler, TVI measures the ***time to S*_*m *_*peak ***(***T***_***s***_) or the ***time to S*_*m *_*onset ***in LV basal and middle segments of the three standard apical views [27,28] (Figure 7). By identifying the presence of one or more differences > 50 ms among regional times of S_m _onset, Ghio and coworkers have demonstrated that intraventricular dyssynchrony is detectable even in 29.5 % of patients with advanced LV dysfunction but normal QRS duration [29]. Using a LV 12-segment model (TVI apical measurements are also unreliable), a ***dyssynchrony index ***(***DI***) can be derived as the standard deviation of the average values of T_s _(***T*_***s***_-*SD***) [27,30] (Figure 8). In his personal experience Yu et al have found that a T_s_-SD of > 32.6 ms predicts inverse LV remodeling after CRT with 100% sensitivity, 100% specificity and 100% accuracy in 30 candidates to CRT [30] (Figure 8).

TSI is an implementation of T_s _method. It displays T_s _in multiple LV segments by colour coding wall motion green (corresponding to early systolic contraction) or red, which corresponds to delayed contraction (sensitivity = 87%, specificity = 81% and accuracy = 84% at a cut-off value of 34.4 ms in 56 patients with severe heart failure) [31].

**Figure 9:**
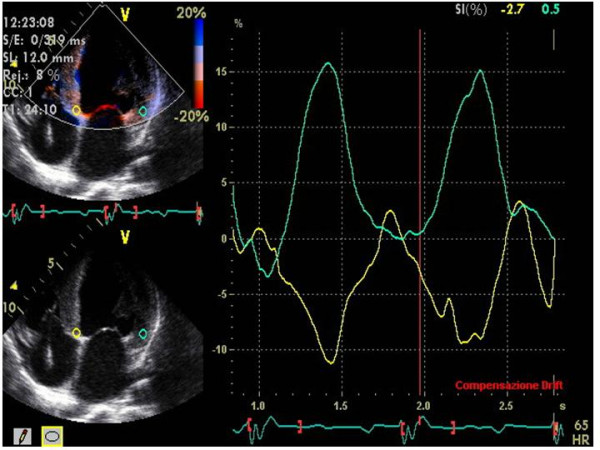
**Strain (%) of basal posterior septum and lateral wall in apical 4-chamber view**. Lateral wall shows an abnormal relaxation (positive sign of its curve) during systole, with a motion that is opposite to that of the basal posterior septum.

**Figure 10:**
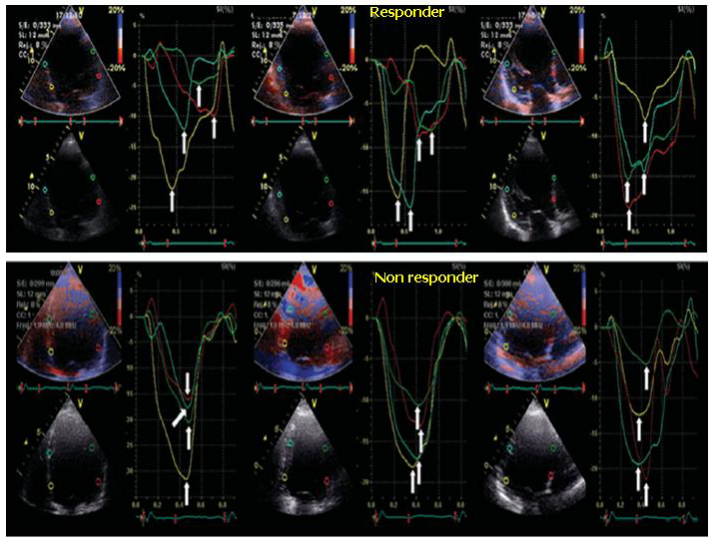
**Longitudinal strain-derived dyssynchrony**. Longitudinal strain-derived dyssynchrony illustrated by 12-TPS-SD (white arrows) evaluated in the three standard apical views (4-, 2- and 5-chambers) before CRT. In a responder patient (upper panel) TPS values are disperse indicating a severe degree of dyssynchrony. In a non responder patient TPS are very close to each other, indicating a low level of intra-ventricular dyssynchrony. (Modified from Mele D et al, *Eur Heart J *2006;27:1070–1078).

**Figure 11:**
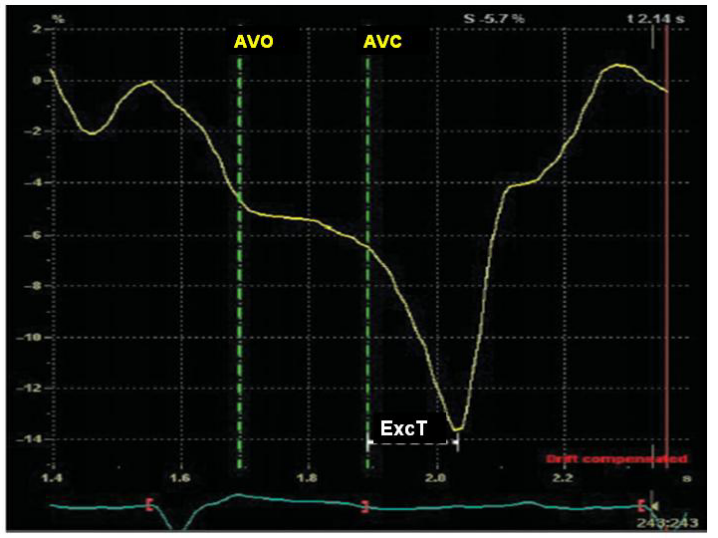
**Methodology for measuring time of exceeding aortic valve closure contraction**. The sum of the time of strain tracing exceeding aortic valve closure (ExcT) is calculated on the 12 LV basal and middle segments in the standard apical views. AVO = aortic valve opening, AVC = aortic valve closure(Modified from Porciani MT et al, *Eur Heart J *2006;27:1818–1823)

Ultrasound instrumentations running Colour Tissue Doppler are also able to determine regional electromechanical delay by means of off-line analysis of longitudinal SRI which, in comparison to TVI, has the advantage of distinguishing active contraction from passive myocardial motion. In general, the detection of intra-ventricular dyssynchrony by means of the strain (%) and strain rate (1/sec) is based on unmasking myocardial "***post-systolic shortening" ***after aortic valve closure (AVC), i.e. during the diastolic myocardial relaxation time (Figure 9). Various methods have been proposed for calculating intra-ventricular dyssynchrony by SRI, some of which (e.g., % of LV base with delayed contraction) predict accurately LV inverse remodeling after CRT but are complicate and requires considerable experience of the operator [32]. One simple method, from Mele and coworkers, measures the ***standard deviation of the averaged time-to-peak-strain ***(***TPS-SD***, ms) of 12 LV basal and mid-segments obtained from the three standard apical views: a TP-SD of > 60 ms is associated with a good response to CRT in 37 patients with dilated cardiomyopathy, although sensitivity, specificity and accuracy have not been determined by this method [33] (Figure 10). Another recent possibility, proposed by Porciani et al, considers the time spent by 12 LV segments in contracting after AVC, i.e., during the isovolumic relaxation time, and measures the ***sum of the time of strain tracing exceeding AVC ***(***ExcT) ***on the 12 LV basal and mid-segments (cut-off value = 760 ms, 93.5% sensitivity and 82.8% specificity) (Figure 11) [34]. This method had the advantage to distinguish "contractile systolic asynchrony", i.e., temporal dispersion of the regional electromechanical delay within the ejective phase, from "diastolic contractile asynchrony", which cannot be detected by analysing the ejection phase of the cardiac cycle alone. Of note, in their experience, Porciani et al have found good sensitivity (= 82%) but poor specificity (= 39%) of TVI-derived T_s_-SD. Although Yu et al demonstrated that SRI-derived post-systolic shortening of 12 LV segments is a good predictor of inverse LV remodeling only for the non-ischemic patients [35], SRI indexes have the conceptual advantages to refer to "true" contraction phenomena while colour TVI is not able to distinguish "active" and "passive" motion of LV segments.

It is important to point out that all colour Tissue Doppler derived techniques require high 2-D frames rates (>90 frames/s) [36] and that 2-D image should be optimized with a narrow sector width that includes the basal and middle segments of opposite LV walls and depth setting that include left ventricle, mitral annulus and the base of the left atrium [37]. Colour Tissue Doppler gain has to be adjusted in order to display myocardial motion clearly. At least 3 cardiac cycles should be recorded during held respiration. Before performing measurements, aortic valve opening (= AVO) and AVC must be marked by means of a previous recorded PW Doppler of LV outflow tract, in order to avoid confusion between systolic (normal) and post-systolic (abnormal) contraction [37].

The 2-D strain (speckle tracking) technique has very recently been used to assess radial dyssynchrony before/after CRT. Speckle tracking has been applied to routine mid-ventricular short-axis images to calculate radial strain from multiple circumferential points averaged to six standard segments and dyssynchrony from timing of peak radial strain has been demonstrated to be correlated with Tissue Doppler measures in 47 subjects [38]. A ***time difference ≥ 130 ms between the radial strain peak of LV posterior wall and anterior septum ***has shown to be highly predictive of an improved EF during follow-up, with 89% sensitivity and 83% specificity [38].

### 3-D Echocardiography

**Figure 12:**
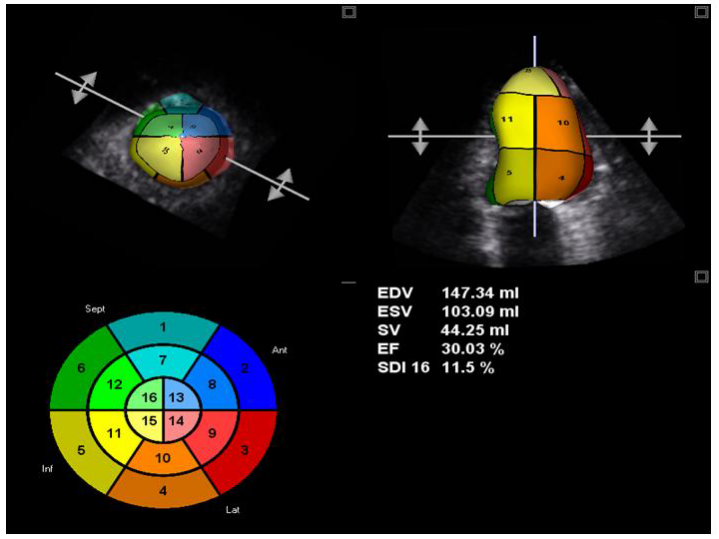
3-D derived time-volume curves of 16-segment model. Presentation 3-D derived time-volume curves of 16-segment LV model in a patient with intra-ventricular dyssynchrony. Each LV myocardial segment is codified by a different colours.

Three-dimensional (3-D) echocardiography allows intra-ventricular dyssynchrony to be evaluated by analyzing LV wall motion in multiple apical planes during the same cardiac cycle. It also offers better spatial resolution than a single plane. The global LV volumetric dataset has been used to determine a dyssynchrony index that corresponds to the standard deviation of the average of the time intervals needed by multiple LV segments to reach minimal end-systolic volume [39] (Figure 12). This index is expressed as the percent value of the overall cardiac cycle, in order to be able to compare patients with different heart rates. CRT responders (= an improvement of NHYA class) show a significant reduction of this 3-D dyssynchrony index, which parallels the reduction of LV end-diastolic volume and the increase in EF [40]. The agreement between 3-D and TVI in identifying the magnitude of intra-ventricular dyssynchrony and the site of maximal delay is poor at only 16% [39]. Advantages of the 3-D method include the possibility of evaluating all LV segments and all of the radial, longitudinal, circumferential elements of LV contraction. The limitations of 3-D method are its suboptimal feasibility (<80%), its temporal resolution of about 40–50 ms, and its inability to distinguish active from passive motion or to make analysis in the presence of atrial fibrillation and/or recurrent premature beats. Furthermore, the index does not yet have a recognized cut-off point.

**Figure 13:**
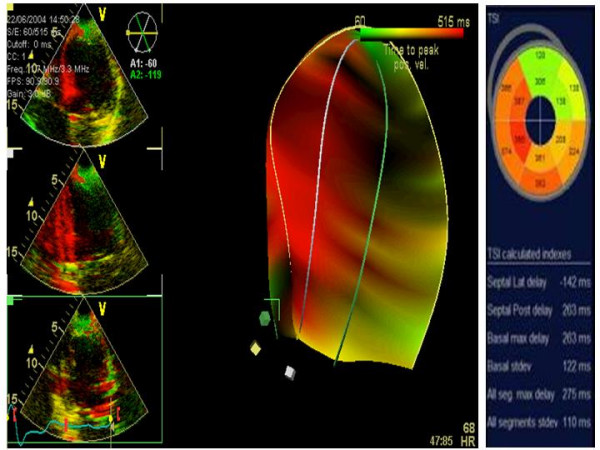
**3-D "surface rendering" TSI for the analysis of LV dyssynchrony**. This approach acquires the 3 standard apical views during the same heart beating (left panels: superior = 4-chamber view; middle = 2-chamber; inferior = 5-chamber) in TSI modality (middle panel). By using this tool several useful parameters can be determined (left panel).

A new "real-time" 3D approach acquires three standard apical views during the same heart beating (Triplane). The images can also be acquired in TSI modality (Figure 13), thus allowing 3-D "*surface rendering*" visualization of the extent of dyssynchrony. The method is fast (a few seconds) and easy to perform, but has a lower temporal resolution than 2-D TSI. Partial experiences from Badano et al [41,42] have pointed out the feasibility and the rapidity of acquisition by using this tool. A very recent study has demonstrated that a ***triplane T*_***s***_-*SD ***(i.e., the standard deviation of time delays in all LV segments calculated by 3-D TSI) = 35.8 ms predicts an acute (48 hours after CRT) reverse LV remodeling with 91% sensitivity and 85% specificity [43].

## What to measure before and after CRT

On the grounds of the multiple mentioned experiences, the ultrasound examination of candidates for CRT requires specific measurements of mechanical dyssynchrony. This can be made by combining PW/CW Doppler and M-mode echocardiography (or PW Tissue Doppler or color Tissue Doppler derived techniques or 3-D imaging). Table 1 lists the cut-off values of the main techniques that predict responders to CRT while Table 2 sumarizes sensitivity, specificity and accuracy, and also confirmatory or controversial data of these techniques. While confirmatory results are shown only by the same researchers who had created the single methods, subsequent studies by other authors have reported conflicting results when using previous validated indexes. It has also to be taken into account that all the proposed methods and techniques have been applied on restricted population sample size and information about the time needed for data acquisition and/or analysis has been rarely reported. In general, intra-ventricular dyssynchrony appears more predictive of CRT response than inter-ventricular dyssynchrony and the global amount of LV dyssynchrony seems to be critical by using different ultrasound techniques: the greater intra-ventricular dyssynchrony, the higher the possibility of significant inverse LV remodeling. When compared to other techniques, 3-D echocardiography has the potential, important advantage to identify the global cardiac dyssynchrony during the same heart beating. After CRT, Doppler echocardiography provides the possibility to evaluate the optimal AV delay and V-V delay setting that maximizes LV systolic function.

**Table 1:** Main ultrasound techniques, parameters and reference values for detection of intra-ventricular dyssynchrony and prediction of LV reverse remodeling.

**Technique**	**Parameter**	**Authors**	**Cut-off point**
M-mode	SPWMD	Pitzalis et al, J Am Coll Cardiol 2002	> 130 ms
M-mode and PW Doppler	LWPSD	Sassone et al, Am J Cardiol 2007	> 1
PW Tissue Doppler	Diff. of T_s _between LV segments	Bax JJ et al, J Am Coll Cardiol 2004	> 65 ms
TVI	T_s_-SD	Yu et al, Am J Cardiol 2003	> 32.6 ms
TSI	T_s_-SD	Yu et al, J Am Coll Cardiol 2005	> 34.4 ms
SRI	TPS-SD	Mele et al, Eur Heart J 2006	> 60 ms
SRI	ExcT	Porciani MC et al, Eur Heart J 2006	> 760 ms
2D radial strain	Time diff. in peak septal wall-to-posterior wall strain	Suffoletto et al, Circulation 2006	≥ 130 ms
3D echo	Triplane T_s_-SD	Van der Veire NR et al, Am J Cardiol 2007	≥ 35.8 *

* acute (48 hours after CRT) response

CW = Continuous wave, ExcT = Sum of time exceeding aortic valve closure, IVMD = Inter-ventricular mechanical delay, LV PEP = Left ventricular pre-ejection period, LWPSD = Lateral wall post-systolic displacement, PW = pulse wave, SPWMD = Septal-to-posterior wall motion delay, SRI = Strain rate imaging, TSI = Tissue synchronization imaging, TVI = Tissue velocity imaging, T_s_-SD = Standard deviation of time to peak S_m_, TPS-SD = Standard deviation of time to peak strain

**Table 2:** Sensitivity, specificity and accuracy, and confirmatory or conflicting data of the main ultrasound techniques presented in Table 1.

**Technique**	**Parameter**	**Sensitivity**	**Specificity**	**Accuracy**	**Confirmatory/conflicting data**
M-mode	SPWMD	100	63	85	Conflicting
M-mode and PW Doppler	LWPSD	-	-	-	-
PW-Tissue Doppler	Diff. of T_s _between LV segments	92	92	-	-
TVI	T_s_-SD	100	100	100	Confirmatory/Conflicting
TSI	T_s_-SD	87	81	84	
SRI	TPS-SD	-	-	-	-
SRI	ExcT	-	-	-	-
2D radial strain	Time diff. peak septal-to-posterior wall strain	89	83	-	-
Triplane echo	T_s_-SD	91	85	-	-

Abbreviations as in Table 1

Although several studies have demonstrated the superiority of ultrasound over QRS duration to assess LV dyssynchrony, there are no conclusive data on prediction of CRT response either using conventional or more advanced echocardiographic technologies. The Cardiac Resynchronization-Heart Failure (CARE-HF) study is the only large randomized and controlled trial that required direct, ultrasound measurement of cardiac dyssynchrony in a subset of patients with mild to moderate QRS enlargement (= 120–149 ms) (5). However, in the CARE-HF study only 92 patients (11%) underwent CRT based on Doppler echocardiographic indexes of myocardial dyssynchrony. Of consequence, the results cannot be considered exhaustive. It is not unexpected, therefore, that the ECG representation of abnormal cardiac conduction still remains as the main criterion to identify patients with dyssynchronous ventricular contraction. Accordingly, no consensus definition of cardiac dyssynchrony exists as yet from the main cardiologic associations [44-47], although several of the mentioned echocardiographic measures appear very promising
